# *IGF-1*, *IGFBP-1*, and *IGFBP-3* Polymorphisms Predict Circulating IGF Levels but Not Breast Cancer Risk: Findings from the Breast and Prostate Cancer Cohort Consortium (BPC3)

**DOI:** 10.1371/journal.pone.0002578

**Published:** 2008-07-02

**Authors:** Alpa V. Patel, Iona Cheng, Federico Canzian, Loïc Le Marchand, Michael J. Thun, Christine D. Berg, Julie Buring, Eugenia E. Calle, Stephen Chanock, Francoise Clavel-Chapelon, David G. Cox, Miren Dorronsoro, Laure Dossus, Christopher A. Haiman, Susan E. Hankinson, Brian E. Henderson, Robert Hoover, David J. Hunter, Rudolf Kaaks, Laurence N. Kolonel, Peter Kraft, Jakob Linseisen, Eiliv Lund, Jonas Manjer, Catherine McCarty, Petra H. M. Peeters, Malcolm C. Pike, Michael Pollak, Elio Riboli, Daniel O. Stram, Anne Tjonneland, Ruth C. Travis, Dimitrios Trichopoulos, Rosario Tumino, Meredith Yeager, Regina G. Ziegler, Heather Spencer Feigelson

**Affiliations:** 1 Department of Epidemiology and Surveillance Research, American Cancer Society, Atlanta, Georgia, United States of America; 2 Department of Epidemiology and Biostatistics, Institute for Human Genetics, University of California San Francisco, San Francisco, California, United States of America; 3 Genomic Epidemiology Group, German Cancer Research Center, Heidelberg, Germany; 4 Epidemiology Program, Cancer Research Center, University of Hawaii, Honolulu, Hawaii, United States of America; 5 Division of Cancer Prevention, National Cancer Institute, Bethesda, Maryland, United States of America; 6 Division of Preventive Medicine, Department of Medicine, Brigham & Women's Hospital, Harvard Medical School, Boston, Massachusetts, United States of America; 7 Core Genotyping Facility, National Cancer Institute, Gaithersburg, Maryland, United States of America; 8 INSERM, Institut Gustave Roussy, Villejuif, France; 9 Program in Molecular and Genetic Epidemiology, Epidemiology Department, Harvard School of Public Health, Boston, Massachusetts, United States of America; 10 Department of Public Health of Guipuzcoa, San Sebastian, Spain; 11 Division of Cancer Epidemiology, German Cancer Research Centre (DKFZ), Heidelberg, Germany; 12 University of Southern California, Los Angeles, California, United States of America; 13 Department of Medicine, Channing Laboratory, Brigham & Women's Hospital and Harvard Medical School, Boston, Massachusetts, United States of America; 14 Institute of Community Medicine, University of Tromsö, Tromsö, Norway; 15 Department of Surgery, Malmö University Hospital, Malmö, Sweden; 16 The Center for Human Genetics, Marshfield Clinic Research Foundation, Marshfield, Wisconsin, United States of America; 17 Julius Center for Health Sciences and Primary Care, University Medical Center Utrecht, Utrecht, The Netherlands; 18 Department of Oncology, McGill University, Montreal, Quebec, Canada; 19 Department of Epidemiology and Public Health, Imperial College, London, United Kingdom; 20 Institute of Cancer Epidemiology, Danish Cancer Society, Copenhagen, Denmark; 21 Cancer Research UK Epidemiology Unit, University of Oxford, Oxford, United Kingdom; 22 Department of Epidemiology, Harvard School of Public Health, Boston, Massachusetts, United States of America; 23 Cancer Registry Azienda Ospedaliera “Civile M.P. Arezzo”, Ragusa, Italy; 24 Division of Cancer Epidemiology and Genetics, National Cancer Institute (NCI), US National Institutes of Health (NIH), Department of Health and Human Services (DHHS), Bethesda, Maryland, United States of America; Innsbruck Medical University, Austria

## Abstract

IGF-1 has been shown to promote proliferation of normal epithelial breast cells, and the IGF pathway has also been linked to mammary carcinogenesis in animal models. We comprehensively examined the association between common genetic variation in the *IGF1*, *IGFBP1*, and *IGFBP3* genes in relation to circulating IGF-I and IGFBP-3 levels and breast cancer risk within the NCI Breast and Prostate Cancer Cohort Consortium (BPC3). This analysis included 6,912 breast cancer cases and 8,891 matched controls (n = 6,410 for circulating IGF-I and 6,275 for circulating IGFBP-3 analyses) comprised primarily of Caucasian women drawn from six large cohorts. Linkage disequilibrium and haplotype patterns were characterized in the regions surrounding *IGF1* and the genes coding for two of its binding proteins, *IGFBP1* and *IGFBP3*. In total, thirty haplotype-tagging single nucleotide polymorphisms (htSNP) were selected to provide high coverage of common haplotypes; the haplotype structure was defined across four haplotype blocks for *IGF1* and three for *IGFBP1* and *IGFBP3*. Specific *IGF1* SNPs individually accounted for up to 5% change in circulating IGF-I levels and individual *IGFBP3* SNPs were associated up to 12% change in circulating IGFBP-3 levels, but no associations were observed between these polymorphisms and breast cancer risk. Logistic regression analyses found no associations between breast cancer and any htSNPs or haplotypes in *IGF1*, *IGFBP1*, or *IGFBP3*. No effect modification was observed in analyses stratified by menopausal status, family history of breast cancer, body mass index, or postmenopausal hormone therapy, or for analyses stratified by stage at diagnosis or hormone receptor status. In summary, the impact of genetic variation in *IGF1* and *IGFBP3* on circulating IGF levels does not appear to substantially influence breast cancer risk substantially among primarily Caucasian postmenopausal women.

## Introduction

The insulin-like growth factor-I (IGF-I) signaling pathway stimulates cell proliferation and inhibits apoptosis [1], [2]. The bioavailability of IGF-I in circulation and tissues is determined by the amount of free ligand that circulates unattached to binding protein. There are six IGF binding proteins. Approximately 75–90% of IGF-I binds to IGFBP-3, limiting its bioavailability. IGFBP-1 also modulates IGF-I bioavailability, and is inversely regulated by insulin [3]. IGF-I has been shown to promote proliferation of normal epithelial breast cells [1], [2], [4]. The IGF pathway has been linked to mammary carcinogenesis in animal models [5], and consequently, it has been extensively examined in relation to breast cancer pathogenesis.

Previous epidemiologic studies have suggested that high circulating levels of IGF-I and low levels of IGFBP-3 are associated with increased risk of premenopausal breast cancer [6], [7]. Numerous recent epidemiologic studies (reviewed in [6]) have begun to examine variation in the genes encoding *IGF1, IGFBP1*, and *IGFBP3* in relation to breast cancer risk. The most extensively examined polymorphisms in *IGF1* has been the 5′ simple tandem repeat that lies 1-kb upstream from the *IGF1* gene transcription start site (the most common allele in Caucasians is the 19 CA repeat) and an A/C polymorphism 5′ to *IGFBP3* at nucleotide −202 (rs2854744) [6]. Some studies report that these or other *IGF* polymorphisms modestly affect circulating levels of IGF-I and IGFBP-3 [6], [8], [9], [10], [11], [12], but there is limited support for a direct effect on breast cancer risk. Most recently, comprehensive analyses of common genetic variation across the *IGF1, IGFBP1*, and *IGFBP3* genes were conducted in two prospective cohorts [8], [9], [11], but no association with breast cancer risk was observed.

To comprehensively examine the role of common genetic variation in the *IGF1, IGFBP1*, and *IGFBP3* genes in relation to circulating IGF-I and IGFBP-3 levels and breast cancer risk, we conducted a haplotype-based analysis in the NCI Breast and Prostate Cancer Cohort Consortium (BPC3) [13]. The large size of this study (cases = 6,912/controls = 8,891) enabled us to detect modest genetic effects, explore gene-environment interactions, and examine potentially important subclasses of tumors, such as those defined by stage or hormone receptor status.

## Methods

### Study Population

The BPC3 has been described in detail elsewhere [13]. Briefly, the consortium includes large well-established cohorts assembled in the United States or Europe that have DNA for genotyping and extensive questionnaire data from cohort members. This analysis includes 6,912 cases of invasive breast cancer and 8,891 matched controls from six cohorts: the American Cancer Society Cancer Prevention Study-II (CPS-II; [14]), the European Prospective Investigation into Cancer and Nutrition (EPIC) cohort [15], the Harvard Nurses' Health Study (NHS; [16]), the Harvard Women's Health Study (WHS; [17]), the Hawaii-Los Angeles Multiethnic Cohort Study (MEC; [18]), and the Prostate, Lung, Colorectal, and Ovarian Cancer Screening Trial cohort (PLCO; [19]). With the exception of MEC, most women in these studies are Caucasian. Written informed consent was obtained from all subjects, and each cohort has been approved by the following institutional review boards: Emory University (CPS-II), International Agency for Research on Cancer (IARC) and each EPIC recruitment center (EPIC), Harvard University (NHS and WHS), University of Hawaii and University of Southern California (MEC), and the U.S. National Cancer Institute and the 10 study screening centers (PLCO).

Cases were initially identified in each cohort by self-report and subsequently verified from medical records or tumor registries and/or linkage with population-based cancer registries. In all cohorts, questionnaire data were collected prospectively before the cancer diagnosis. Controls were matched to cases by age, ethnicity (except in PLCO), and in some cohorts additional matching criteria were utilized (e.g. date of blood draw).

### SNP Selection and Genotyping

The details of *IGF1*, *IGFBP1* and *IGFBP3* characterization and selection of haplotype-tagging SNPs (htSNPs) have been described elsewhere [9], [20]. Briefly, coding regions of *IGF1, IGFBP1*, and *IGFBP3* were sequenced in a panel of 95 advanced breast cancer cases from the MEC (19 from each of the five ethnic groups; African American, Latina, Japanese, Native Hawaiian, and Caucasian). SNPs were also selected from public databases to capture the genetic diversity of regions from ∼20 kb upstream to ∼10 kb downstream of each gene. Haplotype blocks (regions of strong linkage disequilibrium) were defined using the method of Gabriel et al. [21]. Haplotype tagging SNPs (htSNPs) were selected to predict the common haplotypes among Caucasians that meet a criterion of r_h_^2^>0.80.

For genetic characterization of *IGF1*, 154 SNPs were genotyped a multiethnic panel of 349 individuals with no history of cancer *(18)*. Of the 154 SNPs genotyped, 53 were identified as monomorphic and 37 had poor genotyping results (i.e., genotyped ≤75% of samples or out of Hardy-Weinberg equilibrium [one-sided *P*<.01] in more than one ethnic group)—these 90 SNPs were eliminated from further analysis. The remaining 64 SNPs were used for genetic characterization and had an average density of one SNP for every 2.4 kb over a 156-kb region. Fourteen htSNPs were selected using the expectation-maximization algorithm [22] to predict the common haplotypes among Caucasians (r_h_^2^>0.85). For genetic characterization of *IGFBP1* and *IGFBP3* (which are located contiguously in a 35kb region of chromosome 7), 56 SNPs were genotyped in the multiethnic panel *(18)*. Of the 56 SNPs genotyped, 17 were identified as monomorphic and 3 had poor genotyping results (as discussed above)—these 20 SNPs were eliminated from analysis. The remaining 36 SNPs were used for genetic characterization, having an average density of one SNP for every 2 kb over a 71-kb region. Twelve htSNPs were selected to predict the common haplotypes among Caucasians (r_h_^2^>0.99). Additionally, two genic SNPs in *IGFBP3* that were not part of a haplotype block were examined (rs6670, rs2453839), and two additional *IGFBP3* SNPs (rs2132570, and rs2960436) were included. Thus, a total of 16 SNPs across *IGFBP1* and *IGFBP3* were evaluated. Genotyping of breast cancer cases and controls was performed in four laboratories (University of Southern California, Los Angeles, CA USA, Harvard School of Public Health, Boston, MA USA, International Agency for Research on Cancer, Lyon, France, National Cancer Institute Core Genotyping Facility, Gaithersburg, MD USA) using a fluorescent 5′ endonuclease assay and the ABI-PRISM 7900 for sequence detection (Taqman). Initial quality control checks of the SNP assays were done at the manufacturer (ABI, Foster City, CA); an additional 500 test reactions were run by the BPC3. Assay characteristics for the *IGF1*, *IGFBP1*, and *IGFBP3* htSNPs are available on a public website (http://www.uscnorris.com/mecgenetics/CohortGCKView.aspx). To assess interlaboratory variation, each genotyping center ran assays on a designated set of 94 samples from the Coriell Biorepository (Camden, NJ) (22). The completion and concordance rates were each >99%[23]. The internal quality of genotype data at each genotyping center was assessed by typing 5–10% blinded samples in duplicate or greater, depending on study.

### IGF-I and IGFBP-3 Measurements

IGF-I and IGFBP-3 levels were measured by enzyme-linked immunosorbent assays among non-users of postmenopausal hormones (and non-users of oral contraceptives in EPIC). Detailed laboratory methods for these studies have been previously reported [24], [25], [26]. Blood samples analyzed in this study include all cohorts with the exception of the CPS-II and WHS cohorts, where most specimens were collected after diagnosis (CPS-II) or hormone assays were not performed (WHS). Thus, these analyses included 6,410 women for IGF-I and 6,275 women for IGFBP-3.

### Statistical Analysis

In our hormone analyses, circulating IGF-I and IGFBP-3 values were naturally log-transformed to provide approximate normal distributions. Geometric mean levels of IGF-I and IGFBP-3 for *IGF1* and *IGFBP3* SNPs were calculated using linear regression analysis while adjusting for age at blood draw, assay laboratory and batch for circulating IGFs, BMI, race/ethnicity, and country within EPIC cohort. Additional regression analyses were conducted simultaneously adjusted for all other *IGF1* and *IGFBP* SNPs to determine the best fit model of circulating levels.

In our breast cancer analysis, we examined both single SNP and haplotype effects on breast cancer risk. For single SNP analyses, we used conditional multivariate logistic regression to estimate odds ratios (ORs) for breast cancer using a linear (log-odds additive) scoring for 0, 1 or 2 copies of the minor allele of each SNP. For the haplotype analyses, we calculated haplotype frequencies and subject-specific expected haplotype counts separately for each cohort, by country within EPIC, and by ethnicity within the MEC. An expectation-substitution approach was used to assign expected haplotype counts based on unphased genotype data and to account for uncertainty in assignment [27]. The most common haplotype was used as the referent group. Rare haplotypes (those with estimated individual frequencies <5%) were combined into a single category.

To test the global null hypothesis of no association between variation in *IGF1*, *IGFBP1*, or *IGFBP3* haplotypes and risk of breast cancer (or subtypes defined by receptor status), we used a likelihood ratio test comparing a model with additive effects for each common haplotype (treating the most common haplotype as the referent) to the intercept-only model. To test for heterogeneity across cohorts and ethnic groups, we used the Wald *X* ^2^ for the htSNPs and a likelihood ratio test for the haplotypes.

We considered conditional models both without and with adjustment for known breast cancer risk factors. These included menopausal status (premenopausal, postmenopausal), age at menopause (<50, 50+, age unknown), BMI (<25, 25-<30, 30+, missing), parity (ever, never, missing), use of postmenopausal hormones (ever, never, missing), first-degree family history of breast cancer (yes, no, unknown), age at menarche (<13, 13–14, 15+, missing), and use of oral contraceptives (ever, never, missing). Because results remained virtually unchanged regardless of the model used, we present results from the conditional models adjusting for matching factors only. We also evaluated BMI, family history of breast cancer, and use of postmenopausal hormones for possible interaction effects using likelihood ratio testing (LRT). Models with the main effect of genotype and the covariate of interest were compared to models with the main effects of genotype and the covariate of interest, plus a multiplicative interaction term of the two variables. We also examined whether the associations between *IGF1, IGFBP1*, or *IGFBP3* htSNPs or haplotypes and breast cancer differed by menopausal status (pre- versus post-menopausal), stage (*in situ* versus localized versus regional or distant metastasis) or hormone receptor (ER and PR) status.

Lastly, this analysis includes a portion of the previously published data from the MEC [9], [11] and EPIC [8] cohorts (n = 2,522 breast cancer cases). Thus, all associations were examined in sub-analyses that excluded the MEC and EPIC cohort participants.

## Results

**Figure 1:**
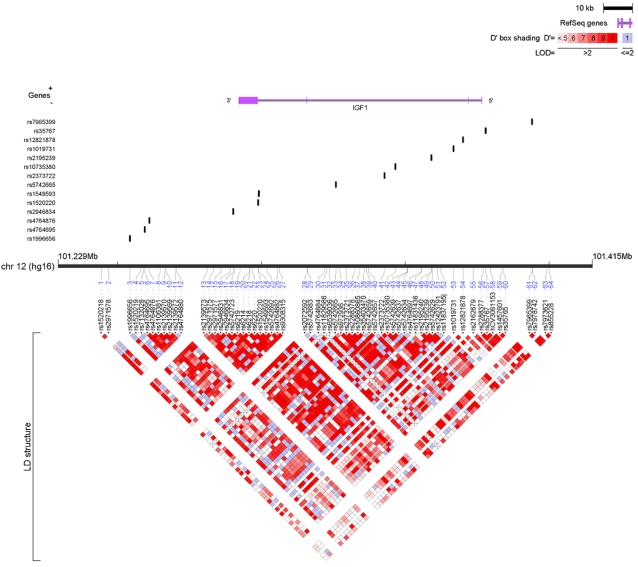
IGF1 SNPs and linkage disequilibrium. 64 SNPs were identified covering a 56-kb region. Of these, 14 htSNPs defined the common haplotypes among Caucasians.

**Figure 2:**
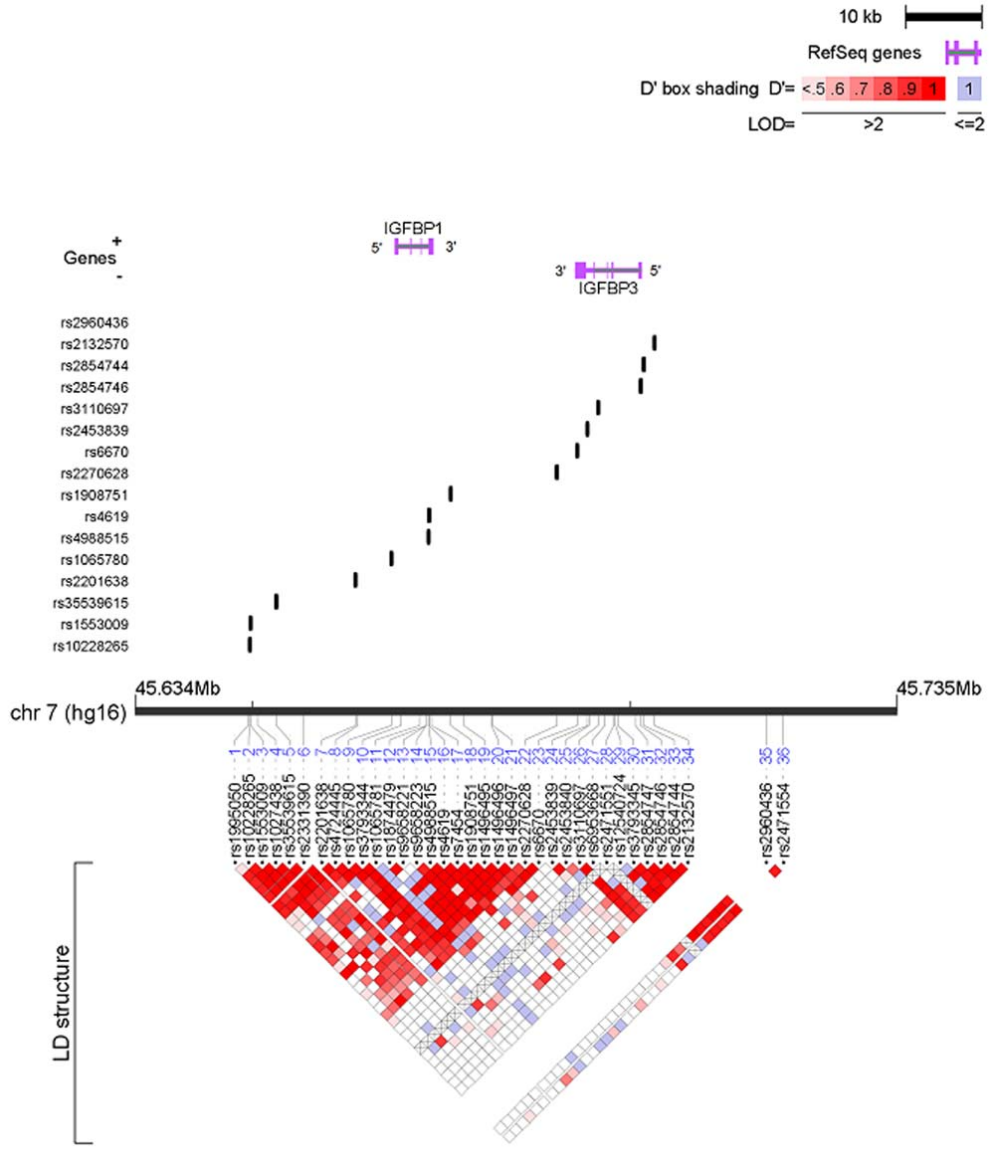
IGFBP1 and IGFBP3 SNPs and linkage disequilibrium. 36 SNPs were identified covering a 71-kb region. Of these, 12 htSNPs defined the common haplotypes among Caucasians.

The genomic structure of *IGF1* is shown in Fig. 1 and that of *IGFBP1* and *IGFBP3* is shown in Fig. 2. The *IGF1* locus was characterized into four haplotype blocks. *IGFBP1* and *IGFBP3* loci are 19kb apart and were characterized by three haplotype blocks. The genotyping success rate was ≥95% for all SNPs at each genotyping center. No deviation from Hardy-Weinberg equilibrium was observed among the controls overall (at the p<0.01 level). The frequencies of individual SNPs and common haplotypes within each LD block were consistent across all cohorts (data not shown).

Study characteristics of each cohort (except PLCO) have been published previously [28]. Briefly, case and control characteristics were comparable across all cohorts and most women were postmenopausal (n = 5,474 cases and 9,732 controls) and Caucasian. As there was no heterogeneity in results across cohorts for any main effects analyses, we only reported results from pooled analyses across all cohorts combined. Additionally, haplotype analyses did not contribute additional information beyond individual SNP results, thus we reported only results for all individual SNPs within each haplotype block.

SNPs in *IGF1* (Table 1) and *IGFBP3* (Table 2) were associated with circulating IGF-I and IGFBP-3 levels, respectively, in women not taking postmenopausal hormones. SNPs in *IGF1* block 1 were most closely associated with circulating levels; the variant alleles were significantly associated with higher circulating IGF-I levels (trend p = 0.0075 for rs7965399 and p = 0.0262 for rs35767). However, these SNPs (wild type vs. variant homozygote) individually accounted for less than a 5% change in mean IGF-I levels. Results did not differ after simultaneously adjusting for all other *IGF1* and *IGFBP* SNPs in the regression analysis (data not shown). The strongest relationships for IGFBP-3 were observed with five SNPs in *IGFBP3* block 3: rs3110697, rs2854746, rs2854744, rs2132570, rs2960436 (trend p<0.001 for all). Rs2854746 remained significantly associated with IGFBP-3 levels (p<0.0001) after adjusting for all other *IGF1* and *IGFBP* SNPs simultaneously in the regression analysis. These SNP associations account for a change in mean circulating IGFBP-3 levels ranging from 6% (rs2132570) to 12% (rs2854746).

**Table 1:** Associations between IGF1 SNPs and mean circulating IGF-I and IGFBP-3 levels in the BPC3.

SNP (position)	Genotype	N (n = 6,410)	mean IGF1 diff	p-trend	% change	N (n = 6,410)	mean IGFBP-3 diff	p-trend	% change
**Block 1**									
rs7965399	TT	5620	26.8		0	5495	122.1		0
(101394153)	TC	530	28.0	0.008	4.4	522	119.9	0.075	−1.8
	CC	41	28.1		4.8	41	112.7		−8.3
rs35767	GG	4184	26.9		0	4100	121.5		0
(101378036)	GA	1704	27.4	0.026	1.8	1662	120.6	0.049	−0.7
	AA	209	27.9		3.7	205	115.3		−5.4
**Block 2**									
rs12821878	GG	3830	27.1		0	3751	121.6		0
(101370134)	GA	2039	26.8	0.187	−1.3	1993	122.1	0.164	0.4
	AA	326	26.8		−1.3	317	125.2		3
rs1019731	CC	4826	27.2		0	4718	121.5		0
(101366892)	CA	1312	27.1	0.915	−0.5	1288	121.4	0.856	−0.1
	AA	103	28.1		3.3	101	123.5		1.6
rs2195239	CC	3754	26.8		0	3673	121.3		0
101359169	CG	2182	27.4	0.028	2.3	2134	121.3	0.932	0
	GG	330	27.2		1.7	326	121.2		0
**Block 3**									
rs10735380	AA	3397	26.8		0	3327	122.1		0
(101346703)	AG	2363	27.5	0.042	2.5	2310	121.5	0.847	−0.5
	GG	454	27.1		1	444	122.6		0.4
rs2373722	GG	5405	27.1		0	5292	121.9		0
(101342924)	GA	809	27.8	0.071	2.4	787	120	0.256	−1.6
	AA	41	27.7		2	42	122.5		0.5
rs5742665	CC	4690	27.1		0	4601	121.6		0
(101326017)	CG	1394	27.3	0.288	0.8	1355	121.1	0.532	−0.4
	GG	120	27.9		3.1	113	119.8		−1.5
rs1549593	GG	4703	27.0		0	4598	121.2		0
(101299258)	GT	1350	26.8	0.754	−0.8	1325	122.7	0.541	1.2
	TT	99	27.7		2.5	95	117.4		−3.2
rs1520220	CC	4068	26.8		0	3976	122.4		0
(101298989)	CG	1902	27.7	0.007	3.1	1868	121	0.157	−1.2
	GG	253	27.2		1.3	249	120.5		−1.6
**Block 4**									
rs2946834	GG	2771	26.5		0	2714	122.7		0
(101290281)	GA	2716	27.4	0.007	3.2	2658	121	0.076	−1.4
	AA	704	27.1		2.2	685	120.5		−1.8
rs4764876	GG	3259	26.9		0	3187	121.5		0
(101261169)	GC	2460	27.2	0.218	1.2	2408	121.1	0.812	−0.3
	CC	461	27.1		1.1	451	121.4		−0.1
rs4764695	GG	1592	27.3		0	1559	121.9		0
(101259580)	GA	3122	27.1	0.190	−0.9	3049	121.2	0.875	0.6
	AA	1529	26.9		−1.6	1500	122.2		0.2
rs1996656	AA	4307	27.00		0	4215	120.5		0
(101254429)	AG	1718	27.2	0.605	0.8	1678	120.9	0.882	0.3
	GG	185	26.9		−0.3	182	119.9		−0.5

**Table 2:** Associations between IGFBP1 and IGFBP3 SNPs and mean circulating IGF-I and IGFBP-3 levels in the BPC3.

SNP	Genotype	N (n = 6,275)	mean IGF1 diff	p-trend	% change	N (n = 6,275)	mean IGFBP-3 diff	p-trend	% change
**Block 1**									
rs10228265	AA	3035	26.9		0	2981	122.5		0
(45649695)	AG	2654	27.3		1.4	2587	121		−1.2
	GG	599	27.3	0.108	1.7	585	120.3	0.083	−1.8
rs1553009	GG	4061	27.1		0	3976	121.5		0
(45649774)	GA	1998	27.6		2	1953	122.1		0.5
	AA	235	26.5	0.285	−2	232	124.4	0.270	2.4
rs35539615	CC	3640	27.2		0	3560	121.4		0
(45653244)	CG	2269	27.1		−0.2	2223	121		−0.3
	GG	327	27.3	0.995	0.6	319	121.5	0.788	0.1
rs2201638	GG	5854	27.2		0	5729	122.1		0
(45663690)	GA	413	27.6		1.7	404	120.2		−1.6
	AA	17	27.7	0.349	2	17	113.1	0.209	−8
rs1065780	GG	2358	27.0		0	2309	122		0
(45668457)	GA	2966	27.3		1.1	2902	121.8		−0.2
	AA	923	27.3	0.262	1.1	903	121.2	0.663	−0.7
**Block 2**									
rs4988515	CC	5753	27.1		0	5625	121.7		0
(45673380)	CT	522	27.2		0.5	515	121.1		−0.5
	TT	19	26.1	0.909	−3.8	19	113.9	0.532	−6.8
rs4619	AA	2662	27.0		0	2608	122.3		0
(45673449)	AG	2839	27.2		0.5	2774	121.3		−0.8
	GG	735	27.5	0.243	1.8	719	122	0.543	−0.2
rs1908751	CC	3113	27.2		0	3047	122.2		0
(45676299)	CT	2586	27.0		−0.7	2526	120.6		−1.3
	TT	560	27.1	0.568	−0.3	552	122.6	0.486	0.3
rs2270628	CC	4121	27.3		0	4038	122.8		0
(45690350)	CT	1850	27.1		−0.6	1804	120.1		−2.2
	TT	239	27.1	0.543	−0.7	234	116.4	0.001	−5.5
**Block 3**									
rs3110697	GG	2203	27.4		0	2145	125.4		0
(45695809)	GA	2929	27.1		−1.1	2877	122.2		−2.6
	AA	1083	27.9	0.392	1.8	1059	115.2	<0.0001	−8.9
rs2854746	GG	2010	27.3		0	1970	114.7		0
(45701425)	GC	2950	27.00		−0.9	2884	123		7.2
	CC	1168	27.2	0.741	−0.1	1139	128.5	<0.0001	12
rs2854744	GG	1586	27.6		0	1550	115.6		0
(45701855)	GT	3226	27.1		−2.1	3169	121.5		5.1
	TT	1427	27.6	0.745	−0.3	1388	127.7	<0.0001	10.5
**Additional SNPs**									
rs6670	TT	3841	27.3		0	3764	121.6		0
(45693034)	TA	2146	26.8		−2.1	2091	120.1		−1.2
	AA	278	26.6	0.022	−2.8	275	125.2	0.935	3
rs2453839	TT	4046	27.3		0	3964	122.3		0
(45694353)	TC	1961	27.2		−0.5	1917	121		−1.1
	CC	250	27.5	0.901	0.9	242	121.4	0.264	−0.7
rs2132570	GG	3948	27.1		0	2855	122.4		0
(45703243)	GT	1972	27.1		−0.2	1933	118.9		−2.9
	TT	291	27.5	0.812	1.3	289	115.3	<0.0001	−6.2
rs2960436	GG	1645	27.6		0	1607	115		0
(45718062)	GA	3074	27.1		−1.7	3021	122.5		6.5
	AA	1547	27.3	0.431	−0.9	1506	127.2	<0.0001	10.6

None of the *IGF1* and *IGFBP3* SNPs associated with circulating IGF-I and IGFBP-3 levels were significantly associated with breast cancer risk (Tables 3 and 4 for *IGF1* and *IGFBP1/3*, respectively), nor were other SNPs or haplotypes consistently associated with risk. When examining these associations among invasive breast cancer only, by stage, or by hormone-receptor status, we did not observe any associations between variation in these genes and disease risk (data not shown). Results did not differ when examining associations separately for pre- and post-menopausal women or when restricting the analysis to only white women (data not shown). No consistent interactions were observed among variants in the *IGF1*, *IGFBP1*, and *IGFBP3* genes with any of the following: first-degree family history of breast cancer, ever oral contraceptive use, use of postmenopausal hormones, and BMI (<25, 25-<30, 30+). We observed no interactions resulting in sub-group associations with disease risk (data not shown).

**Table 3:** Association of tagging SNPs of *IGF1* and breast cancer risk in the BPC3.

SNP	Genotype	Cases (n = 6,912)	Controls (n = 8,891)	OR* (95% CI)	p-trend
**Block 1**					
rs7965399	TT	5668	7214	1.00 (ref.)	
	TC	825	1095	0.94 (0.86–1.02)	
	CC	76	105	0.91 (0.71–1.16)	0.12
rs35767	GG	4230	5359	1.00 (ref.)	
	GA	1876	2468	0.96 (0.90–1.03)	
	AA	251	378	0.87 (0.76–1.01)	0.06
TG		5317	6767	1.00 (ref.)	
TA		855	1148	0.97 (0.91–1.03)	
CG		100	129	1.00 (0.84–1.20)	
CA		390	531	0.92 (0.85–1.00)	0.20
**Block 2**					
rs12821878	GG	4073	5343	1.00 (ref.)	
	GA	2090	2544	1.05 (0.99–1.12)	
	AA	299	400	0.98 (0.85–1.12)	0.38
rs1019731	CC	5092	6639	1.00 (ref.)	
	CA	1404	1688	1.05 (0.98–1.13)	
	AA	97	145	0.87 (0.68–1.10)	0.57
rs2195239	CC	3699	4819	1.00 (ref.)	
	CG	2440	3121	1.00 (0.94–1.06)	
	GG	434	532	1.03 (0.91–1.15)	0.83
GCC		3582	4691	1.00 (ref.)	
GCG		1671	2121	1.01 (0.96–1.06)	
ACC		799	983	1.03 (0.96–1.10)	
AAC		594	757	1.02 (0.95–1.10)	
Haplotype Freq <5%		16	22	0.99 (0.61–1.62)	0.93
**Block 3**					
rs10735380	AA	3658	4716	1.00 (ref.)	
	AG	2502	3088	1.03 (0.97–1.10)	
	GG	425	595	0.93 (0.83–1.05)	0.92
rs2373722	GG	5845	7475	1.00 (ref.)	
	GA	757	978	1.00 (0.92–1.10)	
	AA	26	47	0.82 (0.52–1.30)	0.82
rs5742665	CC	5133	6566	1.00 (ref.)	
	CG	1282	1674	1.01 (0.94–1.09)	
	GG	107	131	1.12 (0.89–1.42)	0.52
rs1549593	GG	4994	6455	1.00 (ref.)	
	GT	1416	1734	1.03 (0.96–1.11)	
	TT	114	146	0.98 (0.78–1.22)	0.57
rs1520220	CC	4048	5277	1.00 (ref.)	
	CG	2207	2707	1.03 (0.97–1.10)	
	GG	329	440	0.96 (0.85–1.10)	0.73
AGCGC		3171	4127	1.00 (ref.)	
AGCGG		322	395	0.99 (0.90–1.09)	
AGCTC		697	858	1.04 (0.96–1.12)	
AGGGC		766	991	1.03 (0.96–1.10)	
GGCGC		434	574	0.99 (0.90–1.09)	
GGCGG		719	893	1.03 (0.96–1.10)	
GACGG		406	541	1.00 (0.91–1.09)	
Haplotype Freq <5%		147	196	0.93 (0.79–1.09)	0.88
**Block 4**					
rs2946834	GG	2857	3673	1.00 (ref.)	
	GA	2898	3705	1.01 (0.95–1.07)	
	AA	845	1054	1.03 (0.94–1.12)	0.61
rs4764876	GG	3315	4230	1.00 (ref.)	
	GC	2560	3395	0.97 (0.92–1.04)	
	CC	643	739	1.08 (0.98–1.20)	0.47
rs4764695	GG	1832	2373	1.00 (ref.)	
	GA	3188	4087	1.02 (0.95–1.09)	
	AA	1541	1977	1.01 (0.93–1.09)	0.81
rs1996656	AA	4512	5753	1.00 (ref.)	
	AG	1773	2320	0.98 (0.91–1.04)	
	GG	199	241	1.04 (0.88–1.23)	0.72
GGAA		2753	3506	1.00 (ref.)	
GGGA		1006	1340	0.96 (0.90–1.02)	
GGGG		429	567	0.97 (0.89–1.07)	
AGAA		395	531	0.96 (0.88–1.06)	
ACGA		1134	1411	1.02 (0.96–1.08)	
ACGG		666	860	0.98 (0.91–1.05)	
Haplotype Freq <5%		279	360	1.01 (0.90–1.13)	0.75

* Adjusted for age, race/ethnicity, and country within EPIC cohort.

**Table 4:** Association of tagging SNPs of *IGFBP1* and *IGFBP3* and breast cancer risk in the BPC3.

SNP	Genotype	Cases (n = 6,912)	Controls (n = 8,891)	OR* (95% CI)	p-trend
**Block 1**					
rs10228265	AA	3112	3962	1.00 (ref.)	
	AG	2765	3637	0.98 (0.92–1.04)	
	GG	646	846	0.98 (0.89–1.08)	0.54
rs1553009	GG	4273	5490	1.00 (ref.)	
	GA	2046	2668	0.99 (0.93–1.05)	
	AA	243	336	0.93 (0.80–1.08)	0.419
rs35539615	CC	2760	4836	1.00 (ref.)	
	CG	2298	3073	0.97 (0.92–1.03)	
	GG	389	473	1.01 (0.90–1.14)	0.63
rs2201638	GG	6118	7831	1.00 (ref.)	
	GA	497	706	0.94 (0.85–1.05)	
	AA	34	35	1.17 (0.81–1.68)	0.53
rs1065780	GG	2441	3083	1.00 (ref.)	
	GA	2999	4080	0.95 (0.89–1.01)	
	AA	1055	1282	1.02 (0.93–1.11)	0.812
AGCGG		1655	2070	1.00 (ref.)	
AGGGG		1564	2045	0.95 (0.90–1.01)	
AACGA		1263	1667	0.95 (0.89–1.01)	
GGCGA		1267	1604	0.98 (0.92–1.04)	
GGCGG		555	748	0.94 (0.87–1.02)	
GGCAG		255	357	0.93 (0.83–1.04)	
AGCGA		52	71	0.88 (0.69–1.13)	
Haplotype Freq <5%		112	119	1.11 (0.94–1.32)	0.30
**Block 2**					
rs4988515	CC	5933	7702	1.00 (ref.)	
	CT	627	782	1.00 (0.91–1.11)	
	TT	27	33	1.06 (0.69–1.63)	0.86
rs4619	AA	2706	3456	1.00 (ref.)	
	AG	2964	3886	0.98 (0.92–1.04)	
	GG	907	1122	1.01 (0.93–1.11)	0.94
rs1908751	CC	3238	4134	1.00 (ref.)	
	CT	2677	3518	0.98 (0.92–1.04)	
	TT	593	758	0.99 (0.89–1.10)	0.57
rs2270628	CC	4198	5472	1.00 (ref.)	
	CT	2031	2606	1.00 (0.94–1.06)	
	TT	276	629	0.99 (0.86–1.14)	0.93
CACC		2274	2926	1.00 (ref.)	
CATC		1907	2477	0.98 (0.94–1.04)	
CGCC		1198	1566	0.98 (0.93–1.04)	
CGCT		901	1143	1.00 (0.94–1.07)	
TGCT		348	434	0.99 (0.91–1.09)	
Haplotype Freq <5%		95	136	0.88 (0.73–1.07)	0.84
**Block 3**					
rs3110697	GG	2241	2919	1.00 (ref.)	
	GA	3032	3967	1.00 (0.94–1.07)	
	AA	1191	1491	1.04 (0.95–1.13)	0.47
rs2854746	GG	2142	2759	1.00 (ref.)	
	GC	2983	3843	1.01 (0.94–1.08)	
	CC	1208	1553	1.01 (0.93–1.10)	0.82
rs2854744	GG	1751	2190	1.00 (ref.)	
	GT	3155	4157	0.96 (0.90–1.03)	
	TT	1581	2011	0.98 (0.91–1.07)	0.69
GCT		2778	3601	1.00 (ref.)	
AGG		2707	3464	1.01 (0.97–1.06)	
GGG		733	948	1.00 (0.93–1.08)	
GGT		382	513	0.95 (0.87–1.05)	
Haplotype Freq <5%		122	155	1.03 (0.87–1.22)	0.80
**Additional SNPs**					
rs6670	TT	4210	5555	1.00 (ref.)	
	TA	2022	2602	1.05 (0.98–1.12)	
	AA	305	356	1.12 (0.97–1.30)	0.05
rs2453839	TT	4301	5516	1.00 (ref.)	
	TC	2012	2650	0.99 (0.93–1.05)	
	CC	274	354	1.01 (0.87–1.16)	0.89
rs2132570	GG	3999	5182	1.00 (ref.)	
	GT	2145	2772	0.99 (0.93–1.06)	
	TT	358	441	1.01 (0.89–1.15)	0.99
rs2960436	GG	1795	2237	1.00 (ref.)	
	GA	3127	4156	0.96 (0.89–1.02)	
	AA	1657	2128	0.98 (0.90–1.06)	0.56

* Adjusted for age, race/ethnicity, and country within EPIC cohort

Across all statistical tests performed in relation to disease status, we observed fewer significant findings than those expected by chance alone (15 findings significant at p<0.05; 40 expected by chance alone). None of these findings provided clear evidence for main effect or subgroup associations for any of the SNPs or common haplotypes. Thus we believe these sporadic associations may reflect chance. Finally, we repeated all analyses excluding subjects from the MEC and EPIC cohorts, and found no meaningful differences in associations when compared to overall findings (data not shown).

## Discussion

Our study is by far the largest to examine genetic variation in the *IGF1*, *IGFBP1*, and *IGFBP3* genes in relation to both circulating IGF-I and IGFBP-3 levels and breast cancer risk. Several genetic variants in *IGF1* and *IGFBP3* predicted circulating levels of IGF-I and IGFBP-3, respectively, but no associations between these variants and breast cancer, overall or in subgroups, were seen. It is thus unlikely that these polymorphisms and their associated hormone levels substantially affect breast cancer risk. There was also no evidence of effect modification by selected breast cancer risk factors or subgroup effects, including menopausal status. While some previous epidemiologic studies have shown stronger support for a role of the IGF-I signaling pathway in premenopausal breast cancer [6], but we did not observe an association among premenopausal women alone.

Our findings are consistent with two previous studies that comprehensively examined the role of *IGF1*, *IGFBP1*, and *IGFBP3* genetic variation in relation to circulating IGF-I and IGFBP-3 levels and breast cancer risk [8], [9], [11]. Cases and controls from these two studies (EPIC and MEC) were included in the pooled analysis. However, sensitivity analyses that excluded these studies also found an association with circulating hormone levels. Other studies have primarily examined individual variants in *IGF1, IGFBP1*, or *IGFBP3* in relation to breast cancer with mixed results [6], [29], [30], [31]. The most extensively studied variant in *IGF1* is the (CA)_n_ repeat polymorphism that lies 1-kb upstream of the *IGF1* transcriptional start site [6], [31]. Some previous studies observed an association between this polymorphism and circulating IGF-I levels (reviewed in [6]); however, most did not observe a corresponding association with breast cancer risk. While we did not genotype *IGF1* (CA)_ n_ polymorphism, we used data from a prior study [24] and determined that the less common repeat length for this polymorphism is in LD with the minor alleles of htSNPs in block 1, rs7965399 and rs35767. Thus, our reported associations with htSNPs in block 1 and circulating IGF-I levels appear consistent with previous literature, that genetic variation influences circulating IGF-I levels, but not at a level substantial enough to impact breast cancer risk.

The A/C polymorphism at nucleotide −202 in *IGFBP3* (rs2854744), and located in haplotype block 3, has been the most extensively examined polymorphism in the IGF binding proteins[6], [8], [9], [29], [30], [31]. Some [6], [29], [30], [31] but not all previous studies [6], [8], [9], [29], [30], [31] have reported an association with breast cancer . This polymorphism has also been associated with circulating levels of IGFBP-3 [26], [30]. Our study confirms the previously reported findings with circulating IGFBP-3 levels, but neither the polymorphism (within Block 3 of *IGFBP3* gene) nor the haplotype block were associated with breast cancer risk in our data.

Strengths of the BPC3 include its size and the comprehensive characterization of variation around the *IGF1*, *IGFBP1*, and *IGFBP3* loci. The latter allows our analysis to provide powerful null evidence against a main effect association between breast cancer risk and variants in these genes that are common among Caucasian women as well as in defined subgroups of the study population.

In summary, results from this large collaborative study support previous evidence that specific genetic variants in *IGF1* and *IGFBP3* genes significantly influence circulating levels of IGF-I and IGFBP-3, respectively, but have no measurable effect on breast cancer risk. Given the large size of our study, it is unlikely that these loci contribute substantially to breast cancer risk among white, primarily postmenopausal, women, at the population level.
